# Making sense of families, households and care

**DOI:** 10.4102/safp.v62i1.5192

**Published:** 2020-12-10

**Authors:** Bernhard M. Gaede

**Affiliations:** 1 Department of Family Medicine, College of Health Sciences, University of KwaZulu-Natal, Durban, South Africa

**Keywords:** family, household, care

## Abstract

The book – *Connected Lives*: *Families, households, health and care in South Africa* (edited by Nolwazi Mkhwanazi and Lenore Manderson)^1^ – is about some of the core business of family medicine. In primary healthcare, and family medicine in particular, the context of the person being treated is central. The evolving understanding of social determinants of health and disease, the linkages between biological illness with social, nutritional, environmental and political context are increasingly important. These shifts require health practitioners to practice differently to how many have been trained traditionally, being challenged to be much more explicit in linking clinical care to the context of the person (‘patient’). Yet, it is this context that clinicians often struggle to understand how the family and households are constituted, how it ‘works’ and what the dynamics are.

‘Connected Lives’^1^ offers a range of perspectives of how the context in the family or household can be understood. Through a series of case studies, the fallacy of the still-dominant idea of the nuclear family is debunked and offers a glimpse into the complexity of how families and households are made and maintained, the impact of illness, how care happens and how we can make meaning of such dynamics.

To quote from Chapter 1: ‘In this book we explore contemporary changes in family-making and household structure, and economic and care arrangements, and we show how household composition and kinship relations influence decisions regarding caregiving and support.’ (page 6)^1^.

The book explores households and families using a ‘life-course’ perspective, starting with how families are made, how they are maintained, how particularly men engage with caring and how illness impacts the family as well as how families age. Within this trajectory many perspectives are explored further, such as gender roles and dynamics, negotiating complex power relations, cultural perspectives and changing social practices over time , and deeply how people make sense of illness, caring and the complex decision-making that this involves.

What makes the book particularly powerful is that it goes well beyond the broad strokes of statistics, income dynamics and disease profiles we are used to from population and public health perspectives. While presenting a big-picture analysis, the book provides a much more nuanced view through the narratives presented. The individual stories paint a picture that (sitting in the consulting room) resonate with the stories we hear daily. It offers ways of thinking and exploring these complexities – whether considering a disability grant application, struggling with the tension between biomedical imperatives and individual or cultural practices or trying to survive in the context of endemic poverty.

The centrality of everyday as a point of departure offers openings and incredible insights for us to understand the dynamics that are often stigmatised, essentialised and flattened into unhelpful rhetoric. Issues such as intimate-partner violence, ‘teenage pregnancy’ or coping with disability or frailty resonate in the voices of the people interviewed (and the views of the researchers presenting them) and these help us to move beyond simplistic and reductionist run-of-the-mill explanations.

The book arose out of several meetings and workshops from 2015 to 2017 where ethnographic and population-based research work from a range of disciplinary backgrounds was presented. It is reflected in the way that the book is written and seeks to capture the deep learning that takes place in such trans-disciplinary spaces. Rather than merely being a collection of chapters by individual researchers presenting their work, it seems to move to a meta-level, where the authors draw case studies from a range of research projects *as told by the researchers* to explore the topic of focus. The effect is interesting – the original quotes of research participants remain intact, yet part of the narrative (and creating a context for this) is the narrative of the researchers – which then is further synthesised and reflected upon by the author of the chapter. Some seemingly small detail can thereby be placed into a context and meta-context and take on much deeper and broader meaning. The book seems to achieve the opposite of what happens in the healthcare system – where the story of a person (in becoming a patient) is abstracted, synthesised and repeatedly re-told (oftentimes inaccurately with increasingly less detail) as the person-now-patient moves through the healthcare system. Unlike the effect in the healthcare system, in the re-telling, the book aims to honour the voice, meaning and context that the person and the story comes from.

While readily accessible, for the biomedically trained clinician the language (and some of the concepts) may seem new and somewhat strange (in a similar way that ‘medical-speak’ is strange to the uninitiated). Yet, it is precisely this language with its lexicon of words and ideas that offers a richness that compliments the daily experience of dealing with people that we come into contact with and perhaps learn to listen and hear with. As such the book is an amazing read for healthcare practitioners, as it helps us to deepen our understanding of the individuals, families, households and communities that we work with and challenges us to pay attention to the stories and the meaning-making of the stories that we hear. Such careful attention is likely to impact on how we manage the illnesses that they present with.

The book is also a rich resource for academic family medicine and primary healthcare. For the teaching of undergraduates, the collection of case studies offers a formidable treasure trove to present complex issues. It can be used as vignettes in lectures or tutorials, reading material for a flipped class-room or incorporated in community-based assignments. For the postgraduates the book offers a deeper exploration of linking clinical care with the social determinants of care. The conceptual positioning of many of the case studies can challenge the biomedical dominance in the discipline and adds to the possible ‘ways of thinking’ for the research of the postgraduate students, from specialization and research masters to PhD’s.

The book is available through academic bookstores.
